# A Systematic Review Comparing the Prognostic Role of eGFR According to CKD-EPI and Older Age Validated Equations in Older Adults

**DOI:** 10.1016/j.xkme.2025.101098

**Published:** 2025-09-09

**Authors:** Elisa K. Bongetti, Benjamin Lazarus, Rory Wolfe, Kevan R. Polkinghorne

**Affiliations:** 1 Department of Nephrology, Monash Medical Centre, Monash Health, Melbourne, Victoria, Australia; 2 Department of Medicine, Monash University, Melbourne, Victoria, Australia; 3 Centre for Health Services Research, University of Queensland, Brisbane, Australia; 4 Department of Nephrology, Princess Alexandra Hospital, Queensland Health, Brisbane, Australia; 5 School of Public Health and Preventive Medicine, Monash University, Melbourne, Victoria, Australia

**Keywords:** Chronic kidney disease, kidney function, older adults, prognosis, glomerular filtration rate

## Abstract

**Rationale & Objective:**

Assessing the clinical relevance of reduced estimated glomerular filtration rate (eGFR) in older adults is challenging because GFR naturally declines with age and not all equations, including the Chronic Kidney Disease Epidemiology Collaboration (CKD-EPI) equations, have been validated for use in older adults. This systematic review compared the association between eGFR and health outcomes in older adults using eGFR equations validated for older age and the CKD-EPI eGFR.

**Study Design:**

Prognostic factor systematic review and meta-analysis.

**Setting & Study Populations:**

Community-dwelling adults aged ≥ 65 years.

**Selection Criteria for Studies:**

Studies using CKD-EPI and at least one of Berlin Initiative Study 1, Berlin Initiative Study 2, European Kidney Function Consortium, Full Age Spectrum, and Revised Lund-Malmo equations to calculate eGFR in association with mortality, cardiovascular outcomes, hospitalizations, or kidney failure. This study was registered on PROSPERO (ID: CRD42022359839).

**Analytical Approach:**

Meta-analysis was undertaken using random-effects models.

**Results:**

Thirteen studies met inclusion criteria with a total of 102,893 participants. The pooled mean age was 80 years (IQR: 76-84), and baseline CKD-EPI_2009_ eGFR was 63 mL/min/1.73m^2^ (IQR: 55-71). The overall quality of data was low, and most studies did not adjust for albuminuria. There was insufficient data assessing the outcomes of cardiovascular outcomes, hospitalizations, or kidney failure. There was no evidence that the association between eGFR and mortality varied according to whether an older age validated equation or CKD-EPI was used. Reduced eGFR according to cystatin C equations had stronger associations with mortality than creatinine-based equations.

**Limitations:**

Limited number of studies and overall low quality of data.

**Conclusions:**

There was no evidence that the association between eGFR and mortality in older adults differed between CKD-EPI and eGFR equations validated for older age. Studies comparing eGFR equations as predictors of hospitalizations, cardiovascular outcomes, or kidney failure were lacking.

## Plain-Language Summary

Assessing the clinical relevance of reduced estimated glomerular filtration rate (eGFR) in older adults is challenging, as GFR naturally declines with age, and not all estimating equations, including the Chronic Kidney Disease Epidemiology Collaboration (CKD-EPI) equations, have been validated for use in older adults. We undertook a systematic review of studies that compared the association between eGFR and health outcomes in older adults using CKD-EPI and older age validated equations, such as the Berlin Initiative Study equations. There was no evidence that the association between eGFR and mortality differed according to whether an older age validated eGFR equation or CKD-EPI was used; however, the quality of the available data was low.

In the general population, the association between chronic kidney disease (CKD) and adverse health outcomes has been well established.^1^ However, in older adults aged ≥ 65 years, the clinical relevance of reduced estimated glomerular filtration rate (eGFR) has been a source of robust debate.^2^^,^^3^ CKD is common in older adults, in part due to physiological decline in GFR and increased burden of cardiometabolic risk factors.^4^ Albuminuria is a sensitive marker of kidney damage and is associated with increased morbidity and mortality, independent of eGFR and beyond what can be attributed to age.^5^, ^6^, ^7^ In the absence of albuminuria; however, it can be challenging to differentiate age-related reductions in eGFR from true disease, with some studies finding no or minimal increase in risk for mortality in older adults with eGFR 45 to 59 mL/min/1.73m^2^.^7^, ^8^, ^9^, ^10^ Additionally, notable differences in the stage and prevalence of CKD vary with the eGFR equation,^11^ which may impact the predictive utility of each equation.

Commonly used eGFR equations, including the Chronic Kidney Disease Epidemiology Collaboration 2009 (CKD-EPI_2009_) and 2021 equations (CKD-EPI_2021_), were formulated from cohorts of generally younger individuals,^12^^,^^13^ and may therefore be less suitable for use in older adults. To address this issue, the Berlin Initiative Study equations (creatinine-based: BIS1 and creatinine and cystatin C-based: BIS2)^14^ were developed specifically for older adults; however, were not listed as a validated equation in the recently updated Kidney Disease: Improving Global Outcomes 2024 CKD clinical practice guidelines.^15^ Other eGFR equations were developed for use across the full age spectrum including older adults (eg, the full age spectrum [FAS] equation,^16^ and the European Kidney Function Consortium [EKFC] equation).^17^ These equations generally perform well in populations of older adults, with improved accuracy in estimating GFR compared with the 2009 CKD-EPI_2009_ equation.^16^^,^^18^^,^^19^ However, it is unclear whether older age validated equations and CKD-EPI differ in their association with long-term health outcomes in older adults. The Kidney Disease: Improving Global Outcomes CKD guidelines recommended that more research should evaluate the difference in performance characteristics of such eGFR equations for outcomes including cardiovascular events and mortality.^15^ Although eGFR equations were not developed to predict long-term health outcomes, studies that aim to relate health outcomes to kidney function serve to assist clinicians in distinguishing physiological age-related variations in eGFR from true, pathological disease.^9^

Therefore, the aim of this study was to undertake a systematic review addressing the utility of older age validated eGFR equations compared with CKD-EPI equations in predicting mortality, cardiovascular outcomes, hospitalizations, and kidney failure in older adults aged ≥ 65 years. Given that the prevalence of CKD in older adults varies considerably depending on the eGFR equation used,^11^^,^^20^ it was hypothesized that there would be differences between equations in the threshold of eGFR at which an increased risk for mortality, cardiovascular events, and hospitalizations was observed, and that older age validated eGFR equations would demonstrate stronger association with health outcomes in older adults than CKD-EPI equations.^21^, ^22^, ^23^, ^24^

## Methods

### Search Strategy

A systematic search was conducted in EMBASE, MEDLINE, and Scopus for studies investigating the association between eGFR and mortality, cardiovascular disease, and hospitalizations using estimating equations validated for use in older adults or across the age spectrum (including older adults) in adults ≥ 65 years. The search period included any studies published until September 2024. A single reviewer (EB) conducted the initial title and abstract screen. Full reports were obtained for studies that appeared to meet inclusion criteria based on title and abstract. Two reviewers (EB and BL) then undertook full-text review. Disagreements were resolved by consensus. Details of the search terms and strategy is shown in the Supplementary Methods. We prospectively registered the systematic review protocol PROSPERO (ID: CRD42022359839). This study was prepared according to PRISMA (preferred reporting items for systematic reviews and meta-analyses) guidelines.^21^

### Selection Criteria

The review question was defined using the PICOTS system (Population, Index prognostic factor, Comparator prognostic factors, Outcome, Timing, Setting), as recommended in guidelines for prognostic factor systematic reviews^22^ (Supplementary Methods). Inclusion criteria were as follows: (1) community-dwelling adults aged ≥ 65 years, (2) creatinine or cystatin C-based baseline eGFR using older age validated equations including the FAS, BIS1, BIS2, and EKFC, (3) creatinine-based CKD-EPI_2009_ or CKD-EPI_2021_, or the cystatin C-based 2012 CKD-EPI equation (CKD-EPI_2012_), and (4) measured outcomes of mortality, or hospitalizations or cardiovascular outcomes or kidney failure.

### Outcomes Assessed

The type of cardiovascular events reported varied between studies and was therefore specified for each applicable study. Where hazard ratios (HRs) or other quantitative measures were provided as an estimate of the prognostic utility of older age validated eGFR equations, these were extracted.

### Data Collection Process

One reviewer (EB) extracted data of included studies into a pre-prepared data collection sheet (Supplementary Methods). A checklist for critical appraisal and data extraction for systematic reviews of prognostic factor studies (CHARMS-PF) provided guidance about which items should be extracted from studies.^22^ Information on participant demographic details, study methodologies, HR, and 95% confidence intervals were extracted.

### Risk of Bias

Two authors (EB and BL) independently assessed each study’s risk of bias using the quality in prognostic factor studies tool.^22^ To assess the overall certainty and quality of summary statistics, the Grades of Recommendation, Assessment, Development and Evaluation (GRADE)^23^ approach was used.^25^ Further information are available in the Supplementary Methods.

### Meta-Analyses

All statistical analyses were undertaken in “R” (R Core Team, 2022). Because of the heterogeneity between studies, a random-effects model with a restricted maximum-likelihood estimator was used to pool study-specific results. The mean ages and baseline eGFR values using the same equations were pooled, and the overall proportion of female participants was calculated.

As most included studies did not report an HR for eGFR modeled as a continuous variable, we estimated the HR trend across categorical data for each eGFR equation and outcome with a method based on generalized least squares using the dosresmeta package.^24^ The average eGFR for each category was estimated by using the midpoints for each category. For unbounded categories such as eGFR <30 or ≥60 mL/min/1.73m^2^, values were chosen based on available information about trial cohorts. Further information is provided in the Supplementary Methods (S1). There was insufficient data available to perform meta-analysis of diagnostic accuracy tests, including receiver operating characteristic area under the curve values.

As a systematic review, informed consent and ethics approval were not required for this study.

## Results

### Characteristics of Selected Studies

Overall, 4,163 articles were identified with our search strategy, and 4,130 were excluded after screening titles and abstracts (Fig S1). The full text of 33 studies was considered, of which 13 met inclusion criteria and are summarized in Table 1.^25^, ^26^, ^27^, ^28^, ^29^, ^30^, ^31^, ^32^, ^33^, ^34^, ^35^, ^36^ Twelve studies used CKD-EPI_2009_ as a comparator, one study used the CKD-EPI_2021_ equation,^34^ and 5 studies additionally used the cystatin C-based CKD-EPI_2012_ equation.^25^^,^^27^^,^^29^^,^^31^^,^^34^

**Table 1:** Characteristics of Included Studies

Author/Y	Country	Setting	Mean Follow-up, y	Participants, n	Age (y), Mean ± SD	Female (%)	Mean Baseline CKD-EPI_2009_ eGFR, SD (mL/min/1.73m^2^)	Diabetes (%)	Hypertension (%)	Older Age Validated Equation/s	Outcomes Measured
Corsonello et al,^27^ 2018	Italy	Community	9	828	74.4 (6.9)	57.1	72 (13.4)	12	63	BIS1, BIS2, and FAS_cr_	Mortality
Van Pottelbergh et al,^25^ 2014	Belgium	Community	2.9	539	84.7 (3.6)	63	61 (19)	19	70	BIS2	Renal Death^a^, CVM, CV events, hospitalizations
Mandelli et al,^28^ 2015	Italy	Community	5	700	89.5 (3.7)	73.3	NS	14	62	MAYO, BIS1	Mortality
Canales et al,^29^ 2017	USA	Community	9	1,289	79.5 (4.6)	100	66.7 (16.7)	NS	62	BIS1, BIS2	Mortality, CVM
Beridze et al,^30^ 2023	Sweden	Community	15	3,094	Median: 72 (IQR 66-81)	63.7	Median: 69 (IQR: 59-78)	8.9	70	RLM^b^, BIS1^b^, and EKFC^b^	Mortality
Canales et al,^31^ 2016	USA	Community	7.3	2,994	76.4 (5.6)	0	69.8 (17.1)	13	63	BIS2	Mortality, CVM
Kuhn et al,^7^ 2021	USA	Community	8.2	1,581	79.9 (6.6)	57.2	NS	25	74	BIS1^b^, BIS2, FAS_cr_^b^, FAS_cr-cys_^b^	Stroke, myocardial infarction, and mortality
Wang et al,^36^ 2020	China	Community	Median: 2.6	278	97 (2)	77	Median: 74 (IQR 62-78)	0	45	BIS1	Mortality
Paparazzo et al,^32^ 2022	Italy	Nursing home	2	522	80.7 (7.8)	68.8	57 (18)	NS	73	BIS1, FAS_cr_	Mortality
Malmgren et al,^33^ 2015	Sweden	Community	NS	1,011	75.2 (0.2)	100	Median: 78 (IQR 20)	7	40	RLM, BIS1	Mortality
Tarantini et al,^26^ 2016	Italy	Outpatient cardiovascular clinic	2	7,845	79 (74-84)	51	NS	30	80	BIS1^b^	Mortality, hospitalizations
Fu et al,^34^ 2024	Sweden	Community	3.9	82,154	77 (8)	50	67 (22)^c^	25	80	EKFC_2023_, EKFC_2021_	Mortality, hospitalizations, kidney failure, and CV events
Bevc et al,^35^ 2018	Slovenia	Unclear	11	58	73 (4.8)	50	40.1 (14)	NS	NS	BIS2, FAS_cr_, FAS_cr-cys_	Mortality

Abbreviations: BIS1, Berlin Initiative Study 1; BIS2, Berlin Initiative Study 2; CKD-EPI_2009_, 2009 creatinine-based Chronic Kidney Disease Epidemiological Collaboration; CVM, cardiovascular mortality; CV, cardiovascular; eGFR, estimated glomerular filtration rate; EKFC_2021_, creatinine-based European Kidney Function Consortium; EKFC_2021_, creatinine-cystatin C-based European Kidney Function Consortium; FAS_cr_, Full Age Spectrum creatinine-based equation; FAS_cr-cys_, Full Age Spectrum creatinine-cystatin C-based equation; IQR, interquartile range; NS, not specified; RLM, Revised Lund-Malmö; SD, standard deviation.

a Renal Death: defined as mortality or the necessity for renal replacement therapy.

b Hazard ratios not provided for individual equation/s as predictors of outcome.

c Calculated with 2021 CKD-EPI equation.

There were a total of 102,893 participants across all studies; 80% of them were from the study by Fu et al.^34^ The pooled mean age across all studies was 80 years (IQR: 76-84), and the pooled proportion of female participants was 74% (IQR: 15-97). Results from meta-analyses on baseline eGFR values according to CKD-EPI, BIS1, and BIS2 are shown in Table 2.^7^^,^^25^, ^26^, ^27^, ^28^, ^29^, ^30^, ^31^, ^32^, ^33^^,^^35^^,^^36^ There was insufficient data available to perform meta-analyses for FAS, EKFC, or other older age validated equations.

**Table 2:** Pooled Baseline Mean eGFRs and Proportions of Participants With eGFR < 60 mL/min/1.73m^2^ according to different estimating equations

	Pooled Value	Studies Used for Meta-Analysis^a^
CKD-EPI_2009_ (Overall)		
Mean CKD-EPI_2009_ eGFR	63 mL/min/1.73m^2^ (IQR: 55-71),	9 studies^25^^,^^27^^,^^29^, ^30^, ^31^, ^32^, ^33^^,^^35^^,^^36^
Proportion <60 mL/min/1.73m^2^ (CKD-EPI_2009_ eGFR)	37% (IQR: 23-55)	8 studies^25^^,^^27^^,^^29^, ^30^, ^31^, ^32^, ^33^^,^^36^
BIS1		
Mean BIS1 eGFR	56 mL/min/1.73m^2^ (IQR: 49-63)	5 studies^27^^,^^30^^,^^32^^,^^33^^,^^36^
Mean CKD-EPI_2009_ eGFR	66 mL/min/1.73m^2^ (IQR: 57-74)	
Proportion < 60 mL/min/1.73m^2^ (BIS1 eGFR)	67% (IQR: 50-81)	7 studies^26^, ^27^, ^28^, ^29^^,^^32^^,^^33^^,^^36^
Proportion < 60 mL/min/1.73m^2^ (CKD-EPI_2009_ eGFR)	37% (IQR: 24-52)	
BIS2		
Mean BIS2 eGFR	58 mL/min/1.73m^2^ (IQR: 46-70)	4 studies^7^^,^^25^^,^^27^^,^^31^
Mean CKD-EPI_2012_ eGFR	65 mL/min/1.73m^2^ (IQR: 45-81)	
Mean CKD-EPI_2009_ eGFR	67 mL/min/1.73m^2^ (IQR: 53-82)	
Proportion < 60 mL/min/1.73m^2^ (BIS2 eGFR)	52% (IQR: 24-79)	4 studies^25^^,^^27^^,^^29^^,^^31^
Proportion < 60 mL/min/1.73m^2^ (CKD-EPI_2012_ eGFR)	31% (IQR: 18-47)	
Proportion < 60 mL/min/1.73m^2^ (CKD-EPI_2009_ eGFR)	34% (IQR: 9-72)	

Abbreviations: BIS1, Berlin Initiative Study 1; BIS2, Berlin Initiative Study 2; CKD-EPI_2009_, 2009 creatinine-based Chronic Kidney Disease Epidemiological Collaboration; CKD-EPI_2012_, 2012 creatinine-cystatin C-based Chronic Kidney Disease Epidemiological Collaboration; eGFR, estimated glomerular filtration rate; IQR, interquartile range.

a Not all studies in this review provided suitable or sufficient data for meta-analysis.

All studies used Cox proportional hazards regression to assess the association between eGFR (calculated with a CKD-EPI equation and at least one older age validated equation) and an outcome. Two studies specified how proportional hazards assumptions were checked.^7^^,^^30^ Three studies assessed discrimination for the association between eGFR and mortality using the C-statistic (Table S1).^27^^,^^30^^,^^32^ Two studies calculated time-dependent area under the receiver operating curve at 2 years,^26^ 15 years,^30^ and one study calculated cumulative area under curve values derived from Cox regression models with 9-year follow-up^30^ (Table S1).^26^^,^^27^^,^^30^ Five studies calculated net reclassification improvement analyses,^7^^,^^26^^,^^29^^,^^31^^,^^36^ and one study calculated cumulative sensitivity and specificity of eGFR by different equations derived from Cox regression models for 9 years follow-up.^27^

Most studies analyzed eGFR as a categorical variable, and the number and bounds of categories varied between studies. Many studies categorized eGFR into 4 or 5 categories, while others used only 2 categories to signify the presence or absence of CKD. The rationale for the eGFR thresholds that defined each category was stated in 4 studies.^7^^,^^26^^,^^30^^,^^33^ There were differences in the referent eGFR category used in survival analyses, with some studies referencing the highest eGFR category (eg: ≥ 75 mL/min/1.73m^2^ or ≥ 90 mL/min/1.73m^2^) and another choosing a middle category as reference (60-89 mL/min/1.73m^2^).^25^ Four studies assessed the potential for a nonlinear relationship between eGFR (as a continuous variable) and mortality using splines.^25^^,^^27^^,^^30^^,^^36^ There was notable variation in the number and choice of variables adjusted for in analyses, and only one study adjusted for albuminuria in analyes^34^ (Table S2).

### Quality of Study Reports

Four studies^26^^,^^32^^,^^35^^,^^36^ were assessed as having a high risk of bias (Table S3). The main limitations were inadequate description of participant response rates and unclear or inappropriate handling of missing data. Nine studies specified the number of participants excluded due to missing data.^7^^,^^25^^,^^28^^,^^29^^,^^31^, ^32^, ^33^^,^^36^ Two studies managed missing data with multiple imputation.^34^^,^^36^ Most studies detailed the setting, inclusion criteria, and follow-up period. In all studies, analyses were based on eGFR values derived from single, cross-sectional measures of serum creatinine or cystatin C.

Evidence for CKD-EPI_2009_ eGFR in association with mortality in older adults was considered low quality, and very low quality for BIS1, BIS2, and FAS (Table S4). Inconsistency in results between studies and imprecision of results were predominant as reasons for downgrading the quality of evidence. There was insufficient evidence to grade the quality of evidence for the remaining eGFR equations as predictors of mortality or for any equations as predictors of hospitalizations, cardiovascular outcomes, or kidney failure.

### Mortality

**Figure 1:**
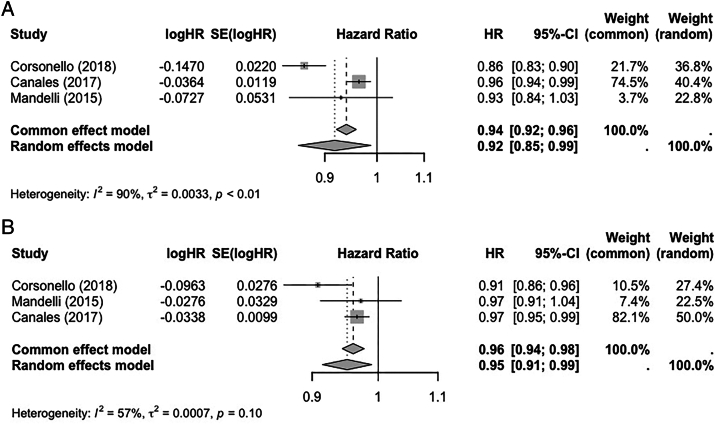
Forest plots showing meta-analyses of adjusted hazard ratios and 95% confidence intervals for the risk of mortality according to estimated glomerular filtration rate (per 5 mL/min/1.73m^2^ increase, calculated using different formulas). (A) Berlin Initiative Study 1 equation. (B) 2009 Chronic Kidney Disease Epidemiological Collaboration equation.

Six studies provided adjusted HRs from survival analyses for the risk of mortality related to baseline BIS1 eGFR (Tables S5a, b).^27^, ^28^, ^29^^,^^32^^,^^33^^,^^36^ Three studies provided sufficient information from survival analyses using BIS1 eGFR to allow meta-analysis.^27^, ^28^, ^29^ The pooled HRs from the random-effects model of the association between BIS1 eGFR and mortality across these 3 studies was 0.92 (95% CI: 0.85-0.99) for every 5 mL/min/1.73m^2^ increase in eGFR using BIS1, compared with 0.95 (95% CI: 0.91-0.99) using CKD-EPI_2009_, as shown in Fig 1. Further details are provided in the Supplementary Results (S1).

**Figure 2:**
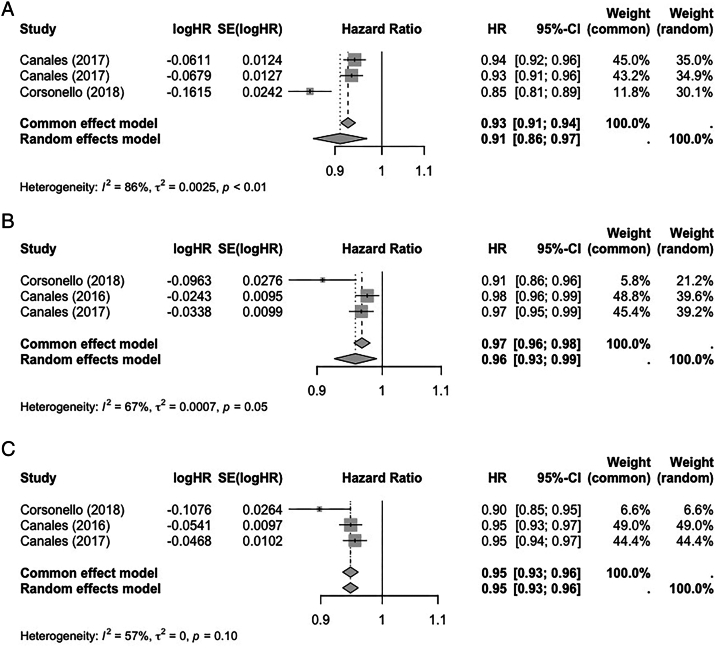
Forest plots showing meta-analyses of adjusted hazard ratios and 95% confidence intervals for the risk of mortality according to estimated glomerular filtration rate (per 5 mL/min/1.73m^2^ increase, calculated using different formulas). (A) Berlin Initiative Study 2 equation. (B) 2009 Chronic Kidney Disease Epidemiological Collaboration equation. (C) 2012 creatinine-cystatin C-based Chronic Kidney Disease Epidemiological Collaboration equation.

Five studies investigated the association of the BIS2 eGFR equation and all-cause mortality (Tables S6a, b),^7^^,^^27^^,^^29^^,^^31^^,^^35^ with 3^27^^,^^29^^,^^31^ providing sufficient data for meta-analysis. Pooled HRs for all-cause mortality were 0.91 (95% CI: 0.86-0.97) for every 5 mL/min/1.73m^2^ increase in eGFR, 0.95 (95% CI: 0.93-0.96), and 0.96 (95% CI: 0.93-0.99) using BIS2, CKD-EPI_2012,_ and CKD-EPI_2009,_ respectively (Fig 2). Reduced eGFR calculated using cystatin C-based equations had stronger associations with mortality than creatinine-based equations (Tables S6a, b; S8).^29^^,^^31^

Four studies assessed the FAS equation for prognostic accuracy.^7^^,^^27^^,^^32^^,^^35^ Two studies provided HRs assessing risk according to eGFR categories (Table S7).^27^^,^^32^ Increased mortality risk was reported for participants with eGFR < 45 mL/min/1.73m^2^ using FAS in one study,^27^ and < 30 mL/min/1.73m^2^ in another study.^32^ Overall, HRs were similar regardless of whether FAS or CKD-EPI_2009_ was used to estimate GFR.^27^^,^^32^^,^^35^

There were limited data on other older age validated equations. Beridze et al^30^ (2023) found Revised Lund-Malmö and EKFC showed better discriminative capacity for predicting mortality compared to CKD-EPI_2009_. In contrast, Malmgren et al^33^ reported the risk of mortality was similar using either Revised Lund-Malmö or CKD-EPI_2009_. Fu et al^34^ found that the association between eGFR and mortality was comparable for creatinine-based 2021 EKFC, CKD-EPI_2021_ and CKD-EPI_2009_^34^ (Table S8).

### Hospitalizations

Van Pottelbergh et al^25^ found the risk of hospitalizations was increased for eGFR < 30 mL/min/1.73m^2^ and was comparable regardless of which equation (BIS2, CKD-EPI_2012_, and CKD-EPI_2009_) was used. Tarantini et al^26^ reported that BIS1 had better accuracy than CKD-EPI_2009_ for predicting the composite outcome of death and hospitalization, based on average net reclassification index of 0.34 (95%CI:0.27-0.38).^26^ Fu et al^34^ found EKFC was a slightly weaker association with hospitalizations compared to CKD-EPI_2009_ and CKD-EPI_2021_.^34^

### Cardiovascular Events

One study found no evidence that BIS1, FAS or CKD-EPI_2009_ eGFR were predictive of cardiovascular mortality.^7^ Three studies reported no association between BIS2 eGFR and cardiovascular events.^25^^,^^29^^,^^31^ Further details are provided in the Supplementary Results (S1).

### Kidney Failure

In a sample of more than 500 Swedish participants aged over 80 years, there was no difference in the risk of kidney failure or death in those with BIS2 eGFR 45-60 mL/min/1.73m^2^ (HR: 1.31, 95% CI: 0.69-2.50) compared with those with an eGFR of 60-90 mL/min/1.73m.^2^^,^^25^ In comparison, risk was increased in those with CKD-EPI_2009_ eGFR 45-60 mL/min/1.73m^2^ (HR: 1.65, 95% CI: 1.05-2.61).

Fu et al^34^ found EKFC and CKD-EPI_2009_ were comparable as predictors of kidney failure requiring replacement therapy, whereas the hazard ratio for the association between CKD-EPI_2021_ eGFR and kidney failure was lower in magnitude (Table S9). The association between cystatin C-based EKFC eGFR and kidney failure was stronger compared with cystatin C-based CKD-EPI_2021_ and CKD-EPI_2012_ eGFR.

### Risk of Publication Bias

Funnel plots suggested no obvious publication bias for the relationship between eGFR (continuous) and mortality using CKD-EPI_2009_, BIS1, or BIS2 (Figs S2-S4); however, there were few studies with sufficient data to include in the plots. There were insufficient studies with data available for meta-analysis to undertake Begg’s or Egger’s tests.^22^

## Discussion

This systematic review aimed to investigate the association between eGFR and health outcomes in older adults, comparing older age validated eGFR equations and CKD-EPI. There was no evidence that the association between eGFR and mortality using CKD-EPI and older age validated eGFR equations differed, and there was insufficient data comparing equations as predictors of cardiovascular outcomes, hospitalizations, or kidney failure. Irrespective of equation, low eGFR < 30 mL/min/1.73m^2^ was associated with mortality. Evidence for an association between moderately reduced eGFR 30-60 mL/min/1.73m^2^ and mortality was mixed, though it appeared reduced eGFR according to cystatin C equations had stronger associations with outcomes than creatinine-based equations. The interpretation of these findings is limited by the low quality of available data, and most studies did not adjust for albuminuria as a potential confounder, which is an important limiting factor. This review highlights the need for future research aimed at evaluating the predictive performance of eGFR equations in older adults to guide appropriate, targeted clinical care in older adults.

Past research has indicated uncertainty with regard to the clinical relevance of mild to moderate reductions in eGFR in older adults, and this issue is further complicated by differences in calculated eGFR between equations and lack of consensus regarding the optimal equation for use in older adults.^2^^,^^8^ In clinical practice, it is challenging to differentiate between true pathological disease from natural, age-related decline in GFR.^37^ While end-stage kidney disease is an obvious consequence of CKD, nonkidney sequelae are perhaps equally relevant to older adults with mild to moderate CKD, in whom the risk for cardiovascular events, hospitalizations, and mortality poses a greater threat than advanced kidney disease.^3^ In this review, there was no evidence that the association between eGFR and health outcomes in older adults differed between equations, but notable heterogeneity existed between studies, which was an important limiting factor.

Between studies, median aged varied from 72 years^30^ to 97 years.^36^ Past research has shown differences in the association between eGFR and mortality in adults aged 65 to 89 compared with those aged ≥ 90 years.^38^ Aging is accompanied by physiological changes and increased burden of chronic disease, which typically accumulates over time, making the comparison of studies with old versus very old participants challenging.^39^ The emergence of age-associated syndromes, including frailty, may lead to overestimation of kidney function using creatinine-based eGFR measures.^40^ Frameworks exist for describing different populations of older adults,^41^ however none of the studies included in our review included these measures, and the majority did not perform analyses on age sub-categories. It is unclear whether differences between study cohorts with respect to chronological age, physical functioning, frailty, or sarcopenia impacted creatinine-based estimations of GFR and risk of death.

Heterogeneity between studies with regard to statistical methodologies is another important consideration. Most authors analyzed eGFR as a categorical variable rather than employing statistical smoothing techniques. As a statistical technique, categorization can lead to loss of information and power.^42^ Additionally, categorization of eGFR limits comparison of results between studies. There was variability between studies in defining eGFR categories and the choice of reference categories used for analyses, which likely contributed significantly to differences seen in results. Spline functions are an alternative approach to modeling nonlinear associations and result in higher explained variance than traditional methods, yet they are not frequently implemented.^43^

There was also significant variability between studies in confounding variables used in adjusted analysis models. Notably, most studies did not adjust for albuminuria, despite strong evidence for an association between albuminuria and mortality and cardiovascular events.^9^ All studies based their analyses on single measures of serum creatinine and did not confirm persistence of kidney function measures over time.

There was a paucity of data available comparing older age validated eGFR equations to CKD-EPI in association with hospitalizations or cardiovascular events. Previous studies have found an association between reduced eGFR and increased incidence of hospitalizations and cardiovascular events in older adults. A study of more than 4,000 adults with a mean age of 76 years found that risk of incident hospitalizations was highest for individuals with eGFR <60 or >90 mL/min/1.73m^2^ using either the creatinine-based CKD-EPI_2009_ or the cystatin C-based CKD-EPI_2012_ equations.^44^ Similarly, analysis of data from the Cardiovascular Health study; a community-based longitudinal study in the United states, found eGFR (using the Modification of Diet in Renal Disease equation) was an independent risk factor for new and recurrent cardiovascular events.^45^

Consistent with previous research,^46^ we found reduced eGFR according to cystatin C-containing equations had stronger associations with outcomes than creatinine-based equations. However, we found insufficient evidence for a difference between cystatin C-containing equations in older adults. Past studies suggest cystatin C-based equations are superior to creatinine eGFR for noncardiovascular deaths in women^47^ and all-cause mortality in both men and women.^48^ International guidelines recommend cystatin C-based eGFR measurement in older adults without additional markers of kidney damage who have moderately reduced eGFR 45 to 59 mL/min/1.73m^2^.^15^ Non-GFR determinants of creatinine and cystatin C are known to impact the associations observed between eGFR and outcomes.^49^ Systemic inflammation, obesity, and being a smoker can result in increased cystatin C, whereas muscle mass and diet can affect serum creatinine.^49^^,^^50^ As many of the non-GFR determinants of increased cystatin C are shared risk factors for cardiovascular disease, it is plausible that previous studies have found cystatin C-based eGFR show strong associations with cardiovascular outcomes.^51^

There were limitations to the review process. First, a single reviewer was responsible for initial title and abstract screen, and data extraction. However, the review methodology was defined a priori and there was consensus between 2 authors in the process of reviewing studies for inclusion and independently assessing for bias. Second, we used the GRADE tool to assess the overall quality of evidence; however, this tool was developed for use in the assessment of interventional studies, and therefore carries limitations when applied prognostic systematic reviews due to increased heterogeneity in study methods and populations.^52^ Third, few studies were included in meta-analyses due to insufficient data, and many of the studies were considered low quality. It would therefore be inappropriate to make strong inferences about the differences in eGFR equations in older adults based on results from meta-analyses. Fourth, most studies undertook survival analyses using eGFR as a categorical variable, however given the heterogeneity of category bounds between studies, overall HRs were derived by estimating the trend across categories to allow for pooling of results. Though accepted as a technique of managing categorical risk data,^24^ it may introduce potential bias. Moreover, as the association between eGFR and mortality may be nonlinear, it is unclear whether the transformation from categorical-based to continuous-based estimates of HRs is appropriate in this instance. Finally, there was insufficient data to accurately assess for publication bias.

## Conclusion

In older community-dwelling adults, the association between eGFR and mortality in older adults was similar when using older age validated equations and CKD-EPI. However, this finding must be interpreted with caution because the quality of available data was low, and most studies did not adjust for urinary albumin in analyses. There was a paucity of research investigating the association between eGFR calculated using older age validated eGFR equations and CKD-EPI and the risk of hospitalizations or cardiovascular outcomes. Given there is notable variation in eGFR produced by differing equations and conflicting findings regarding the associations of reduced eGFR and health outcomes, expert consensus is needed for which equation is optimal for use in older adults. Future studies aimed at comparing CKD-EPI and older age validated eGFR equations as predictors of health outcomes in older adults are required to inform patient-centered care.

## Supplementary Materials

Supplementary File (PDF) Figures S1-S4; Item S1; Tables S1-S9.
