# Supplementary material for: A Systematic Review Comparing the Prognostic Role of eGFR According to CKD-EPI and Older Age Validated Equations in Older Adults
Source: Kidney Med. 2025 Sep 9;7(11):101098. doi: 10.1016/j.xkme.2025.101098 (PMC12595382; doi:10.1016/j.xkme.2025.101098)
Supplement: Supplementary File (PDF) — Figures S1-S4; Item S1; Tables S1-S9. [file mmc1.pdf]

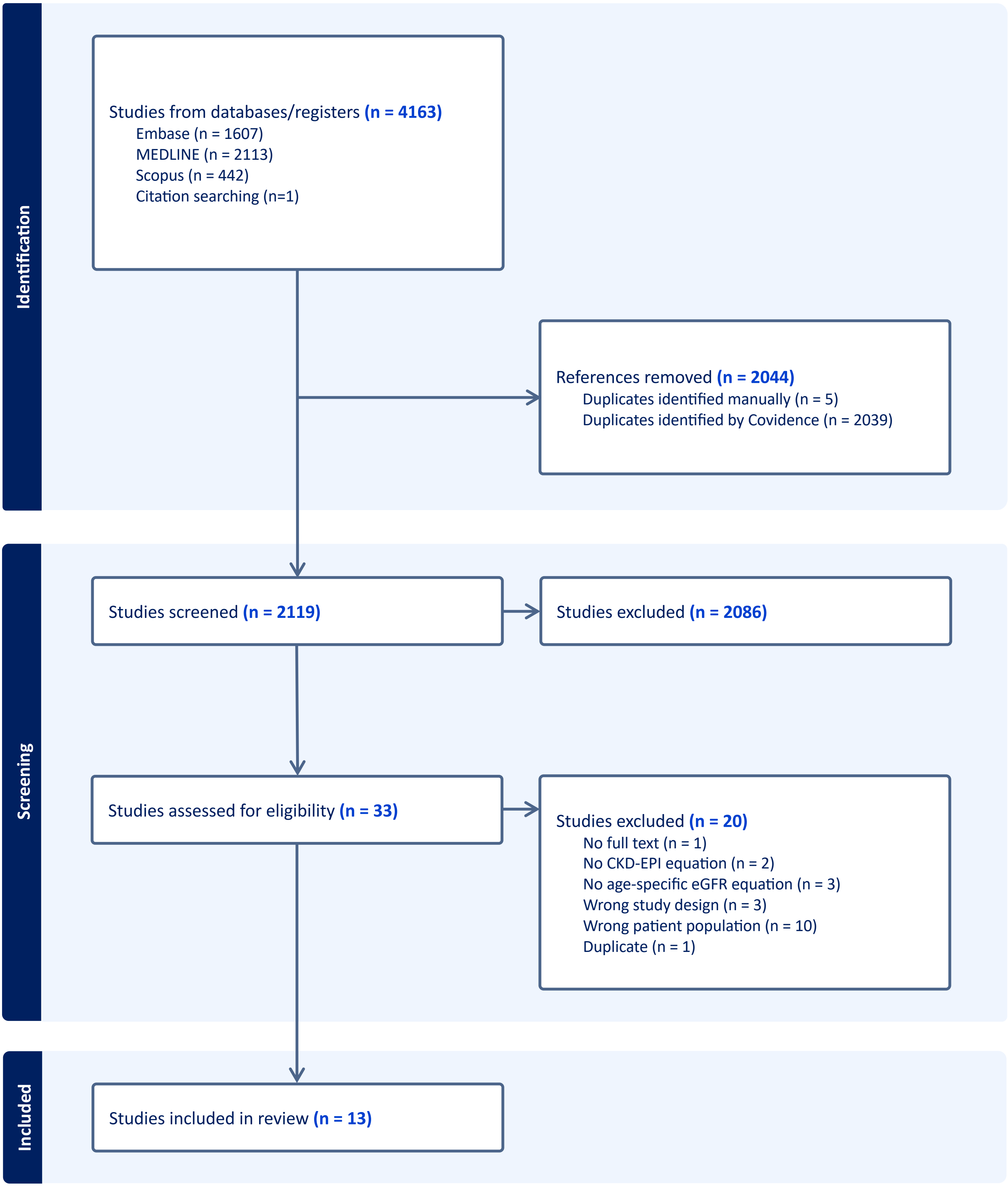

Figure S1: Flow diagram of study screening.

eGFR: estimated glomerular filtration rate; CKD-EPI: Chronic Kidney Disease Epidemiological Collaboration equation

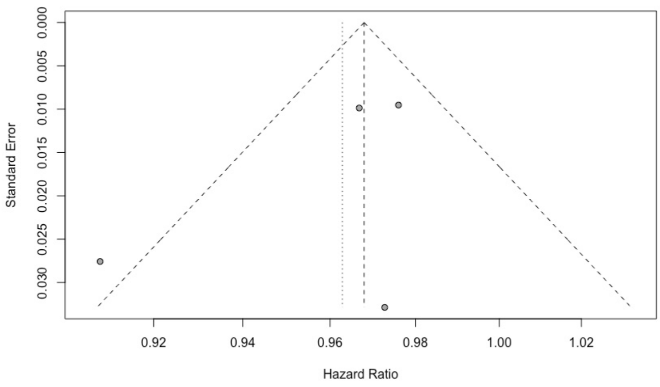

Figure S2: Funnel plot for estimated glomerular filtration rate, calculated using the 2009 creatinine-based Chronic Kidney Disease Epidemiological Collaboration equation, as predictor of mortality

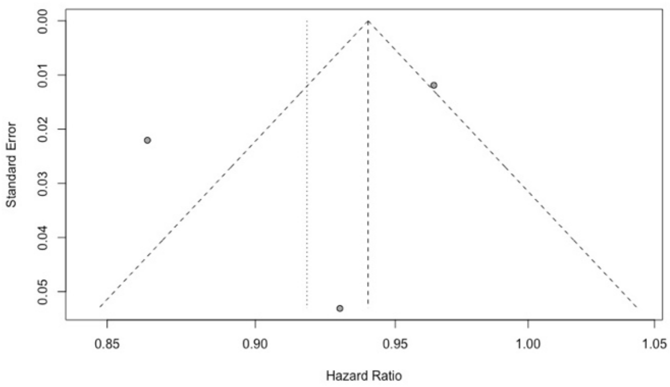

Figure S3: Funnel plot for estimated glomerular filtration rate, calculated using the Berlin Initiative Study 1 equation, as predictor of mortality

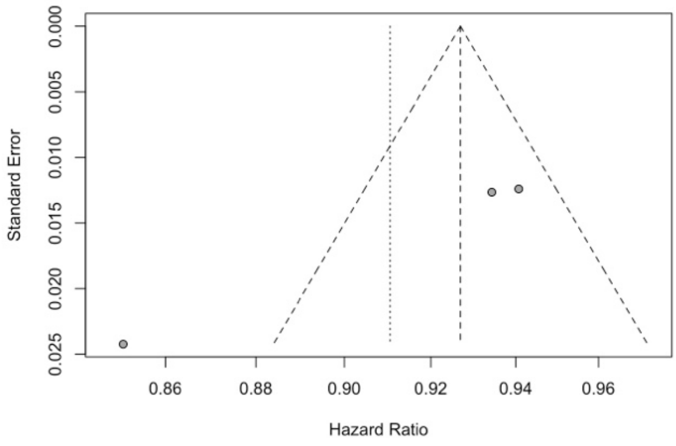

Figure S4: Funnel plot for estimated glomerular filtration rate, calculated using the Berlin Initiative Study 2 equation, as predictor of mortality

Table S1: C-statistic and AUC values (95% confidence intervals) from Cox regression survival models for mortality according to eGFR calculated using different equations.

	Corsonello (2018)*^	Beridze (2023)^	Tarantini (2016)#	Paparazzo (2022)†
**CKD-EPI$_{2009}$**				
C-Statistic	0.61 (0.57–0.65)	0.67 (95% CI: 0.66-0.69)	-	0.79 (0.74-0.85)
AUC	0.60 (0.56-0.64)	0.72 (95% CI: 0.70-0.74)	0.70 (0.68–0.73)	-
**BIS1**				
C-Statistic	0.66 (0.62–0.69)	0.73 (95% CI: 0.72-0.74)	-	0.79 (0.74-0.85)
AUC	0.67 (0.65-0.69)	0.80 (95% CI: 0.78-0.81)	0.70 (0.68–0.72)	-
**FAS**				
C-Statistic	0.64 (0.61–0.68)	-	-	0.79 (0.74-0.84)
AUC	0.63 (0.59-0.67)	-	-	-
**BIS2**				
C-Statistic	0.68 (0.65–0.72)	-	-	-
AUC	0.67 (0.63–0.71)	-	-	-
**EKFC**				
C-Statistic	-	0.70 (0.69-0.71)	-	-
AUC	-	0.76 (0.74-0.77)	-	-
**RLM**				
C-Statistic	-	0.71 (0.70-0.73)	-	-
AUC	-	0.78 (0.76-0.79)	-	-

* For predicting mortality if eGFR<60mL/min/1.73m^2, derived from unadjusted Cox proportional hazards regression model
^ Time point: 15 years
# Time point: 2 years
†Derived from adjusted Cox proportional hazards regression model
AUC: area under the curve, eGFR: estimated glomerular filtration rate; CKD-EPI$_{2009}$: 2009 creatinine-based Chronic Kidney Disease Epidemiological Collaboration, BIS1: Berlin Initiative Study Equation 1 creatinine-based equation, BIS2: Berlin Initiative Study Equation 2 creatinine-cystatin C-based equation, FAS: creatinine-based Full Age Spectrum, EKFC: European Kidney Function Consortium, RLM, Revised Lund- Malmö, FAS: creatinine-based Full Age Spectrum

Table S2: Covariates used in fully adjusted Cox regression survival analyses

Study	Adjustment factors
Corsonello (2018)	Body mass index, smoking habits, hypertension, heart failure, coronary artery disease, stroke, peripheral arterial disease, chronic obstructive pulmonary disease, diabetes, anaemia, femur fracture, osteoarthritis, cancer, Mini-Mental State Examination, basic activities of daily living, Centre for Epidemiological Studies-Depression scale, and muscle cross-sectional area measured at 66% of tibial length.
Mandelli (2015)	Age groups, sex, smoke (current smoker, former smoker), body mass index (≥30 or <18.5), diabetes, hypertension, myocardial infarction, heart failure, stroke, cancer
Paparazzo (2022)	Age at the recruitment, sex, activities of daily living, nutritional status, and Cumulative Illness Rating Score.
Malmgren (2015)	Diabetes, cardiovascular disease, smoking and treatment for high blood pressure
Wang (2020)	Age, gender, marital status, education, smoking, activities of daily living, body mass index, hypertension, cardiovascular disease, cerebrovascular disease, chronic obstructive pulmonary disease, systolic blood pressure, serum albumin, and serum low-density lipoprotein cholesterol.
Van Pottelbergh (2014)	Age, gender, hypertension, diabetes mellitus, history of a serious cardiovascular event and smoking status.
Beridze (2023)	Age, sex, education, diabetes, heart failure, cancer, hypertension, smoking, BMI, and calf circumference
Tarantini (2016)	Sex, prior myocardial infarction, heart failure, peripheral artery disease/stroke, diabetes and persistent/permanent atrial fibrillation
Kuhn (2022)	Age, sex, systolic blood pressure, pulse pressure, self-reported antihypertensive treatment, renin-angiotensin-aldosterone system inhibitor intake, statin intake, diabetes, waist-to-hip ratio, log-transformed C-reactive protein level, total cholesterol level, low-density lipoprotein cholesterol level, and current smoking
Canales (2016)	Age, race, body mass index, hypertension, diabetes mellitus, history of coronary heart disease, and self-reported health status
Canales (2017)	Age, race, body mass index, hypertension, diabetes mellitus, history of coronary heart disease, and self-reported health status
Fu (2024)	For analyses assessing risk of mortality or kidney failure: Age, sex, hypertension, diabetes, cardiovascular disease, and log-transformed urine albumin-to-creatinine ratio<br>For hospitalisations, cardiovascular mortality or events: Age, sex, hypertension, diabetes, cardiovascular disease, and log-transformed urine albumin-to-creatinine ratio, total and high-density lipoprotein cholesterol levels and antihypertensive medication use
Bevc (2019)	Not specified

Table S3: Risk of bias assessed using the Quality In Prognosis Studies (QUIPS) tool.

	Corsonello	Van Pottelbergh	Mandelli	Canales 2017	Beridze	Canales 2016	Kuhn	Wang	Paparazzo	Malmgren	Tarantini	Fu	Bevc
**Author 1: Risk of bias assessment: QUIPS**													
Study participation	Medium	Low	Medium	Medium	Medium	Medium	Low	Medium	High	Low	Medium	Low	High
Study attrition	Medium	Low	Medium	Low	Medium	Low	Low	High	High	High	Unclear	Medium	High
Prognostic factor measurement	Medium	Medium	Medium	Medium	Medium	Medium	Medium	Medium	Low	Medium	Medium	Low	Low
Outcome measurement	Low	Low	Medium	Low	Low	Low	Low	Low	Low	Low	Unclear	Low	Low
Adjustment for other prognostic factors	Low	Medium	Low	Low	Low	Low	Low	Low	Low	High	Medium	Low	High
Statistical analysis and reporting	Low	Medium	Medium	Medium	Medium	Medium	Medium	Low	Low	Medium	High	Low	High
**Risk of bias**	**LOW**	**LOW**	**LOW**	**LOW**	**LOW**	**LOW**	**LOW**	**HIGH**	**HIGH**	**HIGH**	**HIGH**	**LOW**	**HIGH**
**Author 2: Risk of bias assessment: QUIPS**													
Study participation	Low	Low	Low	Low	Low	Low	Low	High	Medium	Low	High	Medium	High
Study attrition	Low	Low	Low	Low	Low	Medium	Low	Medium	Medium	High	Unclear	Low	Medium
Prognostic factor measurement	Medium	Medium	Medium	Low	Medium	Low	Low	Medium	Medium	Medium	Medium	Low	Low
Outcome measurement	Low	Low	Low	Low	Low	Low	Low	Low	Low	Low	Medium	Low	Low
Adjustment for other prognostic factors	Low	Low	Unclear	Low	Low	Low	Low	Low	Low	Low	Low	Low	High
Statistical analysis and reporting	Low	Low	Low	Low	Low	Low	Low	Low	Low	Low	Low	Low	High
**Risk of bias**	**LOW**	**LOW**	**LOW**	**LOW**	**LOW**	**LOW**	**LOW**	**HIGH**	**UNCLEAR**	**LOW**	**HIGH**	**LOW**	**HIGH**
**Overall judgement of risk of bias**	**LOW**	**LOW**	**LOW**	**LOW**	**LOW**	**LOW**	**LOW**	**HIGH**	**HIGH**	**HIGH**	**HIGH**	**LOW**	**HIGH**

Table S4: Quality of evidence for estimated glomerular filtration rate equations as predictors of mortality, assessed using the Grades of Recommendation, Assessment, Development, and Evaluation (GRADE) tool.

Author	$CKD\text{-}EPI_{2009}$	BIS1	BIS2	FAS
**Author 1: GRADE**				
Risk of bias	Not serious	Moderate	Not serious	Not serious
Inconsistency	Serious	Serious	Serious	Serious
Imprecision	Serious	Serious	Very serious	Very serious
Indirectness	Not serious	Not serious	Not serious	Very serious
Publication bias	Unclear	Unclear	Unclear	Unclear
Upgrading factors	None	None	None	None
**Quality of evidence**	**Low**	**Very low**	**Very low**	**Very low**
**Author 2: GRADE**				
Risk of bias	Not serious	Serious	Not serious	Not serious
Inconsistency	Moderate	Serious	Serious	Serious
Imprecision	Serious	Serious	Serious	Very serious
Indirectness	Not serious	Not serious	Not serious	Not serious
Publication bias	Unclear	Unclear	Unclear	Unclear
Upgrading factors	None	None	None	None
**Quality of evidence**	**Low**	**Very low**	**Low**	**Very low**
**Overall quality of evidence**	**LOW**	**VERY LOW**	**VERY LOW**	**VERY LOW**

$CKD\text{-}EPI_{2009}$: 2009 creatinine-based Chronic Kidney Disease Epidemiological Collaboration, BIS1: Berlin Initiative Study Equation 1 creatinine-based equation, BIS2: Berlin Initiative Study Equation 2 creatinine-cystatin C-based equation, FAS: creatinine-based Full Age Spectrum

Table S5a: Adjusted hazard ratios and 95% confidence intervals from studies for all-cause mortality using the Berlin Initiative Study 1 and CKD-EPI equations to calculate estimated glomerular filtration rate.

**eGFR** (mL/min/1.73m^2)	**Canales (2017)**	
	BIS1	CKD-EPI$_{2009}$
**≥75**	1	1
**60–74**	0.9 (0.7-1.1)	1.1 (0.9-1.3)
**45–59**	1.0 (0.8-1.3)	1.1 (0.9-1.4)
**<45**	1.5 (1.1-2.0)	1.7 (1.3-2.2)

eGFR: estimated glomerular filtration rate, CKD-EPI$_{2009}$: 2009 creatinine-based Chronic Kidney Disease Epidemiological Collaboration, HR: hazard ratio, CI: confidence interval
Cells in grey indicate statistically significant hazard ratios

Table S5b: Adjusted hazard ratios and 95% confidence intervals for all-cause mortality using the Berlin Initiative Study 1 and CKD-EPI equations to calculate estimated glomerular filtration rate.

<table>
<tr><th rowspan="2">eGFR (mL/min/1.73m^2)</th><th colspan="2">Corsonello (2018)</th><th colspan="2">Mandelli (2015)</th><th colspan="2">Paparazzo (2022)</th><th colspan="2">Malmgren (2015)</th><th colspan="2">Wang (2020)</th></tr>
<tr><th>BIS1</th><th>CKD-EPI$_{2009}$</th><th>BIS1</th><th>CKD-EPI$_{2009}$</th><th>BIS1</th><th>CKD-EPI$_{2009}$</th><th>BIS1</th><th>CKD-EPI$_{2009}$</th><th>BIS1</th><th>CKD-EPI$_{2009}$</th></tr>
<tr><td>**≥90**</td><td>1</td><td>1</td><td rowspan="2">1</td><td rowspan="2">1</td><td rowspan="2">1</td><td rowspan="2">1</td><td rowspan="2">1</td><td rowspan="2">1</td><td rowspan="2">1</td><td rowspan="2">1</td></tr>
<tr><td>**60–89**</td><td>4.3 (0.6–32.5)</td><td>1.6 (0.8–3.2)</td></tr>
<tr><td>**45–59**</td><td>7.6 (1.0–58.3)</td><td>2.5 (1.2–5.2)</td><td>1.3 (0.87-2.0)</td><td>1.3 (0.9-1.7)</td><td>2.0 (1.0-4.1)</td><td>1.6 (0.8-3.0)</td><td>1.1 (0.8-1.4)</td><td>1.4 (1.0-1.9)</td><td rowspan="3">0.95 (0.7-1.3)</td><td rowspan="3">1.6 (1.2-2.2)</td></tr>
<tr><td>**30–44**</td><td>13.1 (1.6–108.2)</td><td>5.4 (1.1–27.7)</td><td>1.6 (1.06–2.5)</td><td>1.3 (0.9-1.8)</td><td>1.7 (0.8-3.5)</td><td>1.4 (0.7-2.8)</td><td>3.1 (2.0-5.0)</td><td>3.5 (2.1-5.8)</td></tr>
<tr><td>**<30**</td><td>25.3 (2.4–271.9)</td><td>7.42 (1.8–30.6)</td><td>2.5 (1.37-4.4)</td><td>2.0 (1.2-3.3)</td><td>3.8 (1.6-8.9)</td><td>2.4 (1.1-5.0)</td><td colspan="2">NA</td></tr>
</table>

eGFR: estimated glomerular filtration rate, CKD-EPI$_{2009}$: 2009 creatinine-based Chronic Kidney Disease Epidemiological Collaboration, BIS1: Berlin Initiative Study Equation 1 creatinine-based equation HR: hazard ratio, Cells in grey indicate statistically significant hazard ratios.

Table S6a: Adjusted hazard ratios and 95% confidence intervals for all-cause mortality using the Berlin Initiative Study Equation 2 and CKD-EPI equations to calculate estimated glomerular filtration rate.

eGFR (mL/min/1.73m^2)	Van Pottelbergh (2014)*			Corsonello (2018)			Kuhn (2021)^
	BIS2	CKD-EPI$_{2009}$	CKD-EPI$_{2012}$	BIS2	CKD-EPI$_{2009}$	CKD-EPI$_{2012}$	BIS2
**≥90**	4.8 (1.1-21.3)	3.0 (0.9-10.0)	1.1 (0.5-2.9)	1	1	1	1
**60–89**	1	1	1	2.0 (0.6-6.5)	1.6 (0.8–3.2)	1.9 (1.1–3.2)	
**45–59**	1.3 (0.7-2.5)	1.7 (1.1-2.6)	1.3 (0.7-2.3)	4.5 (1.4-14.8)	2.5 (1.2–5.2)	2.3 (1.2–4.3)	1.2 (0.9-1.5)
**30–45**	2.0 (1.1-2.5)	1.7 (1.0-2.9)	1.8 (1.1-3.1)	5.6 (1.5-20.4)	5.4 (1.1–27.7)	5.6 (2.5–12.6)	1.6 (1.2-2.1)
**<30**	5.6 (2.8-11.0)	5.0 (2.9-8.6)	4.3 (2.4-7.6)	13.4 (2.4-75.7)	7.4 (1.8–30.6)	8.9 (2.3–35.0)	

*This study evaluated time to death or renal failure
^Data for CKD-EPI not available
eGFR: estimated glomerular filtration rate, HR: hazard ratio, CI: confidence interval, CKD-EPI$_{2009}$: 2009 creatinine-based Chronic Kidney Disease Epidemiology Collaboration, CKD-EPI$_{2012}$: 2012 creatinine-cystatin C-based Chronic Kidney Disease Epidemiological Collaboration, BIS2: Berlin Initiative Study Equation 2 creatinine-cystatin C-based equation

Table S6b: Adjusted hazard ratios and 95% confidence intervals for all-cause mortality using the Berlin Initiative Study Equation 2 and CKD-EPI equations to calculate estimated glomerular filtration rate.

**eGFR** (mL/min/1.73m^2)	**Canales (2017)**			**Canales (2016)**		
	BIS2	CKD-EPI$_{2009}$	CKD-EPI$_{2012}$	BIS2	CKD-EPI$_{2009}$	CKD-EPI$_{2012}$
**≥75**	1	1	1	1	1	1
**60–74**	1.1 (0.8-1.5)	1.1 (0.9-1.3)	1.1 (0.9–1.3)	1.1 (0.8–1.4)	1.0 (0.9–1.2)	1.2 (1.0–1.5)
**45–59**	1.4 (1.0-1.9)	1.1 (0.9-1.4)	1.3 (1.0–1.6)	1.3 (0.9–1.7)	1.1 (0.9–1.3)	1.3 (1.1–1.6)
**<45**	2.0 (1.4-2.8)	1.7 (1.3-2.2)	1.8 (1.4–2.3)	2.1 (1.5–2.8)	1.5 (1.2–2.0)	2.1 (1.6–2.7)

eGFR: estimated glomerular filtration rate, CKD-EPI$_{2009}$: 2009 creatinine-based Chronic Kidney Disease Epidemiological Collaboration, CKD-EPI$_{2012}$: 2012 creatinine-cystatin C-based Chronic Kidney Disease Epidemiological Collaboration, BIS2: Berlin Initiative Study Equation 2 creatinine-cystatin C-based equation, HR: hazard ratio, CI: confidence interval
Cells in grey indicate statistically significant hazard ratios.

Table S7: Adjusted hazard ratios and 95% confidence intervals for all-cause mortality using the Full Age Spectrum equation and the CKD-EPI equation to calculate estimated glomerular filtration rate.

**eGFR** (mL/min/1.73m^2)	**Corsonello (2018)**		**Paparazzo (2022)**	
	FAS	CKD-EPI$_{2009}$	FAS	CKD-EPI$_{2009}$
**≥90**	1	1	1	1
**60–89**	1.74 (0.52–5.79)	1.63 (0.84–3.17)		
**45–59**	3.31 (0.98–11.1)	2.50 (1.21–5.15)	1.55 (0.79-3.04)	1.48 (0.9-2.45)
**30–45**	4.51 (1.22–16.6)	5.44 (1.10–27.7)	1.44 (0.73-2.84)	0.83 (0.45-1.53)
**<30**	10.3 (1.85–57.9)	7.42 (1.79–30.6)	2.35 (1.10-5.03)	2.37 (1.27-4.41)

eGFR: estimated glomerular filtration rate, CKD-EPI$_{2009}$: 2009 creatinine-based Chronic Kidney Disease Epidemiological Collaboration, FAS: creatinine-based Full Age Spectrum
Cells in grey indicate statistically significant hazard ratios.

Table S8: Adjusted hazard ratios and 95% confidence intervals for all-cause mortality in adults aged ≥65 years using the EKFC equation and CKD-EPI equations to calculate estimated glomerular filtration rate. Values derived from adjusted cubic spline models for specific eGFR values, using mean values of co-variates in the study population.

**eGFR** (mL/min/1.73m^2)	**Fu (2024)**					
	Creatinine-based equations			Cystatin C-containing equations		
	$EKFC_{2021}$	$CKD\text{-}EPI_{2009}$	$CKD\text{-}EPI_{2021}$	$EKFC_{2023\text{-}cys}$	$CKD\text{-}EPI_{2021\text{-}cyscr}$	$CKD\text{-}EPI_{2012\text{-}cys}$
**80**	1	1	1	1	1	1
**60**	0.8 (0.8–0.9)	1.0 (1.0–1.1)	1.0 (0.9–1.0)	1.5 (1.4-1.6)	1.2 (1.1-1.3)	1.3 (1.2-1.4)
**45**	0.9 (0.9–1.0)	1.2 (1.2– 1.3)	1.1 (1.1–1.2)	2.0 (1.9-2.1)	1.4 (1.3-1.4)	1.7 (1.6-1.8)
**30**	1.2 (1.2–1.3)	1.6 (1.5–1.8)	1.5 (1.4–1.5)	2.7 (2.5-2.8)	1.7 (1.7-1.8)	2.1 (2.0-2.2)

eGFR: estimated glomerular filtration rate, EKFC2021: 2021 creatinine-based European Kidney Function Consortium equation, $CKD\text{-}EPI_{2009}$: 2009 creatinine-based Chronic Kidney Disease Epidemiological Collaboration, $CKD\text{-}EPI_{2021}$: 2021 cystatin C-based Chronic Kidney Disease Epidemiological Collaboration, $EKFC_{2023\text{-}cys}$: 2023 cystatin C-based European Kidney Function Consortium equation, $CKD\text{-}EPI_{2021\text{-}cyscr}$: 2021 creatinine-cystatin C-based Chronic Kidney Disease Epidemiological Collaboration, $CKD\text{-}EPI_{2012\text{-}cys}$: 2012 cystatin C-based Chronic Kidney Disease Epidemiological Collaboration
Cells in grey indicate statistically significant hazard ratios.

Table S9: Adjusted hazard ratios and 95% confidence intervals for kidney failure with replacement therapy in adults aged ≥65 years using EKFC equations and CKD-EPI equations to calculate estimated glomerular filtration rate. Values derived from adjusted cubic spline models for specific eGFR values, using mean values of co-variates in the study population.

**eGFR** (mL/min/1.73m^2)	**Fu (2024)**					
	Creatinine-based equations			Cystatin C-containing equations		
	$EKFC_{2021}$	$CKD\text{-}EPI_{2009}$	$CKD\text{-}EPI_{2021}$	$EKFC_{2023\text{-}cys}$	$CKD\text{-}EPI_{2021\text{-}cyscr}$	$CKD\text{-}EPI_{2012\text{-}cys}$
**80**	1	1	1	1	1	1
**60**	1.8 (1.0-3.3)	3.4 (1.2-9.4)	1.4 (0.7-2.8)	2.5 (1.0-6.6)	2.6 (1.2-5.8)	1.5 (0.6-3.9)
**45**	9.2 (5.9-14.5)	10.9 (5.2-23.2)	7.0 (4.3-11.3)	13.1 (5.7-30.0)	8.9 (4.8-16.5)	7.9 (3.8-16.5)
**30**	32.5 (21.3-49.7)	31.3 (15.1-64.9)	23.7 (15.0-37.3)	39.2 (17.4-88.4)	24.9 (13.8-44.8)	16.0 (7.9-32.5)

eGFR: estimated glomerular filtration rate, EKFC2021: 2021 creatinine-based European Kidney Function Consortium equation, $CKD\text{-}EPI_{2009}$: 2009 creatinine-based Chronic Kidney Disease Epidemiological Collaboration, $CKD\text{-}EPI_{2021}$: 2021 cystatin C-based Chronic Kidney Disease Epidemiological Collaboration, $EKFC_{2023\text{-}cys}$: 2023 cystatin C-based European Kidney Function Consortium equation, $CKD\text{-}EPI_{2021\text{-}cyscr}$: 2021 creatinine-cystatin C-based Chronic Kidney Disease Epidemiological Collaboration, $CKD\text{-}EPI_{2012\text{-}cyscr}$: 2012 creatinine-cystatin C-based Chronic Kidney Disease Epidemiological Collaboration
Cells in grey indicate statistically significant hazard ratios.

Item S1: Supplementary Methods and Results

EXAMPLE SEARCH STRATEGY **15**

REVIEW QUESTION: PICOTS **16**

DATA COLLECTION SHEET **17**

META-ANALYSIS OF eGFR VARIABLE **19**

RISK OF BIAS ASSESSMENT **20**

ASSOCIATION BETWEEN EGFR AND MORTALITY **21**

ASSOCIATION BETWEEN EGFR AND CARDIOVASCULAR EVENTS **22**

Example Search Strategy

MEDLINE Search – Ovid interface
exp Aged/
    OR elderly.ti,ab,kf.
    OR older adult.ti,ab,kf.
    OR geriatric.ti,ab,kf.
    OR older person.ti,ab,kf.
AND Glomerular Filtration Rate/
    OR Glomerular Filtration Rate.ti,ab,kf.
    OR ((eGFR or GFR or age-specific or age-adapted or LMR or EKFC) adj4 (equation* or OR estimat*)).ti,ab.
    OR Berlin initiative study*.ti,ab,kf.
    OR BIS1.ti,ab,kf.
    OR BIS2.ti,ab,kf.
    OR (BIS* adj3 (equation* or formula*)).ti,ab,kf.
    OR full age spectrum.ti,ab,kf.
    OR lund-malmo revised.ti,ab,kf.
    OR european kidney function consortium.ti,ab,kf.
AND CKD-EPI*.ti,ab,kf.
    OR chronic kidney disease epidemiology.ti,ab,kf.
AND Hospitalization/
    OR hopitali?ation.ti,ab,kf.
    OR exp Heart Diseases/
    OR heart disease*.ti,ab,kf.
    OR cardiovascular event*.ti,ab,kf.
    OR mortality/ or fatal outcome/ or mortality, premature/
    OR mortality.ti,ab,kf.
    OR Death/
    OR death.ti,ab,kf.
    OR Kidney Failure, Chronic/
    OR kidney failure.ti,ab,kf.
    OR renal failure.ti,ab,kf.
OR end-stage kidney disease*.ti,ab,kf.

Review question: PICOTS

The PICOTS (Population, Index prognostic factor, Comparator prognostic factors, Outcome, Timing, Setting) structure for the systematic review question is shown below:

Population	Index factors	Comparator	Outcomes	Timing	Setting
Older adults aged ≥65 years	Age-specific eGFR equations (BIS1, FAS, EKFC)	Non age specific equations eg CKD-EPI equation, MDRD	Mortality/ Cardiovascular events/ Hospitalisation s/ Secondary: renal failure (eGFR <15 or dialysis)	Timing of baseline prognostic factors: baseline. Follow-up time: no limit	Outpatient/ community-based settings

Data Collection Sheet

**Study Information**
Author
Year
Title
Country
Journal
Source of data<br>e.g., cohort, case-control, or registry data
Start date (dd-mm-yyyy)
End date (dd-mm-yyyy)
Mean follow-up period (years)
Number of centres
Setting
Aim

**Participants**	
Inclusion criteria	
Exclusion criteria	
Age	Mean
	SD
Gender	% Female
Smoking	% Never
	% Past
	% Current
Diabetes	%
Hypertension	% Hypertensive
Dyslipidaemia	%
eGFR (CKD-EPI)	Mean
	SD
UACR	median
	IQR
BMI	Mean
	SD
Cognitive impairment	%
Ischaemic Heart Disease	%
Stroke	%

**Outcomes**	
Definition and method for outcomes measured	
Was the same outcome definition (and method for measurement) used in all participants?	
Types of outcomes (eg, single or combined endpoints)?	
**Prognostic factors**	
Number and type of prognostic factors (eg, obtained from demographics, patient history, physical examination, additional testing, disease characteristics)	
Definition and method for measurement of prognostic factors	
Timing of prognostic factor measurement (eg: at patient presentation, diagnosis, treatment initiation)	
Handling of eGFR (continuous or categorical)	
**Sample size**	
Number of participants analysed	
Number of outcomes	death
	cardiac events
	hospitalisations
	renal failure
**Missing data**	
Number of participants with ANY missing data	
Number of participants with missing eGFR data	
Handling of missing data	(e.g., complete-case analysis, imputation, or other methods)
**Analysis**	
Modelling method (eg, linear, logistic, Cox, parametric survival, competing risks) regression)	
How modelling assumptions were checked; in particular, for time-to-event outcomes and the analysis of hazard ratios, the method for assessing non-proportional hazards (non-constant hazard ratios over time)	
Method for selection of prognostic factors for inclusion in multivariable modelling (eg, all candidate prognostic factors considered, preselection of established prognostic factors, retain only those significant from univariable analysis)	
Method for selection or exclusion of prognostic factors (including those of interest and those used as adjustment factors) during multivariable modelling (eg, backward or forward selection, or full model approach including all factors regardless), and criteria used for any selection or exclusion (eg, P value, Akaike information criterion)	
Method of handling each continuous prognostic factor (eg, dichotomisation, categorisation, linear, non-linear), including values of any cut-points used and their justification; for non-linear trends, the method of identifying non-linear relationships (eg, splines, fractional polynomials)	

Interpretation of presented results	
Comparison with other studies, discussion of generalisability, strengths and limitations	

**Results**	
**eGFR equation(s)**	**Specify the equation(s) used**
**Mortality**	**Unadjusted HR (95%CI)**
	≥90
	60–89.9
	45–59.9
	30–44.9
	<30
	**Unadjusted continuous HR (95%CI)**
	**Adjusted HR (95%CI)**
	≥90
	60–89.9
	45–59.9
	30–44.9
	<30
	Set of adjustment factors used
	C statistic
	**Adjusted HR (95%CI) continuous eGFR**
	death NRI CKD-EPI compared to BIS1
	death NRI BIS1 compared to CKD-EPI
	NRI BIS2 compared to CKD-EPIcre-cys
	AUC
**Hospitalisations**	Unadjusted HR (95%CI)
	Adjusted HR (95%CI)
	Set of adjustment factors used
**CV events**	Unadjusted HR (95%CI)
	Adjusted HR (95%CI)
	Set of adjustment factors used
**Interpretation and Discussion**	

Meta-analysis of eGFR variable

The trend across categorical data was approximated by using the midpoint of estimated glomerular filtration rate (eGFR) categories, or, for unbounded categories, estimates were chosen (shown below):

**Unbounded eGFR category** (mL/min/1.73m^2)	**Midpoint chosen** (mL/min/1.73m^2)
**≥90**	100
**≥75**	90
**≥60**	80
**<45**	30
**<30**	25

The average eGFR for each category was estimated by using the midpoints for each category. This method could only be applied to studies where the number of cases and participants for each eGFR category was provided. Where studies did not report raw data, attempts were made to contact authors to request necessary data to undertake the meta-analyses. Transformed hazard ratios corresponding to the same eGFR equation and outcome variable across studies were pooled using random effects based on log hazard ratios and their standard errors. To assess for publication bias, funnel plots were visually inspected for asymmetry. There was insufficient data for formal, statistical assessment for publication bias.

Risk of Bias Assessment

The quality in prognostic factor studies (QUIPS) tool has six domains that include study participants, study attrition, prognostic factor measurement, outcome measurement, adjustment for other prognostic factors and statistical analysis techniques.

Grades of Recommendation, Assessment, Development and Evaluation (GRADE) considers five domains om rating down estimates of effect: risk of bias, imprecision, inconsistency, indirectness and publication bias. Two authors, (EB and BL) rated the overall strength of evidence for each outcome as ‘high’, ‘moderate’, ‘low’ or ‘very low’.[1]

Association between eGFR and mortality

eGFR calculated with BIS1

The risk of mortality was predicted to be higher for reduced eGFR according to the BIS1 compared to $CKD\text{-}EPI_{2009}$ in three studies.[2–4] In contrast, two studies reported a high risk of mortality for participants with lower eGFR according to CKD-EPI than BIS1.[5–7] Using either BIS1 or $CKD\text{-}EPI_{2009}$ to calculate eGFR, Canales *et al*. (2017) reported the risk of death increased when eGFR was < 45 mL/min/1.73m^2 (BIS1 HR: 1.5, 95%CI: 1.1-2.0; $CKD\text{-}EPI_{2009}$ HR: 1.7, 95%CI: 1.3, 2.2) compared to ≥ 75 mL/min/1.73m^2.[5] In a study very old age adults, Wang *et al.* (2020) found no association between BIS1 eGFR <60 mL/min/1.73m^2 and mortality, compared to eGFR ≥ 60mL/min/1.73m^2, whereas $CKD\text{-}EPI_{2009}$ eGFR < 60 mL/min/1.73m^2 conferred increased risk of death (HR: 1.6, 95%CI: 1.2-2.2).[7] Paparazzo *et al*. (2022) found risk of mortality was increased in older nursing home residents with eGFR <30 mL/min/1.73m^2 using either $CKD\text{-}EPI_{2009}$ or BIS1 (BIS1 HR 3.8, 95%CI:1.6-8.9; CKD-EPI HR: 2.4, 95%CI: 1.1-5.0), but not 30-45 or 45-59 mL/min/1.73m^2, compared to eGFR ≥60 mL/min/1.73m^2.[4]

There was conflicting evidence for the risk of mortality associated with preserved eGFR ≥90mL/min/1.73m^2 using BIS1.[2,8] Beridze *et al*. (2023) modelled the relationship between eGFR and mortality using cubic splines.[8] They identified a non-linear relationship between BIS1 eGFR and death, such that the risk appeared to be higher for eGFR >90 mL/min/1.73m^2 and <40 mL/min/1.73m^2. Based on area under the curve receiver operating characteristic curves and Harrel C statistics obtained from crude logistic regression and Cox regression models, the authors concluded that BIS1 showed the best accuracy for mortality prediction over the 15-year follow-up period, although the prognostic accuracy decreased in participants aged over 77 years.[8] Corsonello *et al.* (2018) also concluded that BIS1 was superior to CKD-EPI for predicting mortality.[2] However, they found no increased risk of mortality for older adults with preserved kidney function (eGFR ≥ 90 mL/min/1.73m^2) using BIS1 or $CKD\text{-}EPI_{2009}$.[2] Tarantini *et al.* (2016) found that compared to $CKD\text{-}EPI_{2009}$, BIS1 was more accurate for mortality prediction in older adults with heart disease based on net reclassification index (NRIs), which were derived from adjusted Cox regression models (average NRI: 0.12; 95% CI: 0.03–0.19).[9]

Association between eGFR and cardiovascular events

Van Pottelbergh *et al.* (2014) reported no evidence of an association between eGFR categories (<30, 30-45, 45-60, 60-90, >90 mL/min/1.73m^2) and the risk of severe cardiovascular events when using either BIS2, CKD-EPI$_{2012}$ or CKD-EPI$_{2009}$ equations to estimate GFR. Additionally, cardiovascular mortality was increased when eGFR <30 mL/min/1.73m^2 using BIS2, CKD-EPI$_{2012}$ or CKD-EPI$_{2012}$ equations, and for CKD-EPI$_{2009}$ eGFR 30-45 mL/min/1.73m^2.[10] Canales *et al.* (2017) found an association between eGFR <45 mL/min/1.73m^2 and cardiovascular mortality when using CKD-EPI$_{2012}$ and CKD-EPI$_{2009}$, but not BIS1 or BIS2.[5] Kuhn *et al.* (2021) found no association between BIS2 eGFR and myocardial infarctions for eGFR 45-59 or <45 mL/min/1.73m^2 relative to eGFR ≥ 60 mL/min/1.73m^2.[11] There was no difference in net reclassification improvement through eGFR using either BIS1, BIS2, FAS, CKD-EPI$_{2009}$ or CKD-EPI$_{2012}$ compared with a basic predictive model that included traditional risk factors for myocardial infarction, including albuminuria.[11] Fu *et al.* (2024) found no difference between creatinine-based EKFC and CKD-EPI$_{2021}$ or CKD-EPI$_{2009}$, and no difference between creatinine-cystatin C-based EKFC, CKD-EPI$_{2021}$ or CKD-EPI$_{2012}$ as predictors of the composite outcome of myocardial infarction or stroke.[12]

Supplementary References

1. Foroutan F, Guyatt G, Zuk V, et al. GRADE Guidelines 28: Use of GRADE for the assessment of evidence about prognostic factors: rating certainty in identification of groups of patients with different absolute risks. *J Clin Epidemiol*. 2020;121:62-70. doi:10.1016/j.jclinepi.2019.12.023
2. Corsonello A, Pedone C, Bandinelli S, Ferrucci L, Antonelli Incalzi R. Predicting survival of older community-dwelling individuals according to five estimated glomerular filtration rate equations: The InChianti study. *Geriatr Gerontol Int*. 2018;18(4):607-614. doi:10.1111/ggi.13225
3. Mandelli S, Riva E, Tettamanti M, Detoma P, Giacomin A, Lucca U. Mortality Prediction in the Oldest Old with Five Different Equations to Estimate Glomerular Filtration Rate: The Health and Anemia Population-based Study. *PLoS One*. 2015;10(8):e0136039-. doi:https://dx.doi.org/10.1371/journal.pone.0136039
4. Paparazzo E, Geracitano S, Lagani V, et al. Clinical and Prognostic Implications of Estimating Glomerular Filtration Rate by Three Different Creatinine-Based Equations in Older Nursing Home Residents. *Front Med (Lausanne)*. 2022;9((Paparazzo, Geracitano, Passarino, Montesanto) Department of Biology, Ecology and Earth Sciences, University of Calabria, Rende, Italy(Lagani) Institute of Chemical Biology, Ilia State University, Tbilisi, Georgia(Lagani) Biological and Environmental Scie):870835. doi:https://dx.doi.org/10.3389/fmed.2022.870835
5. Canales MT, Blackwell T, Ishani A, et al. Renal Function and Death in Older Women: Which eGFR Formula Should We Use? *Int J Nephrol*. 2017;2017:1-10. doi:10.1155/2017/8216878
6. Malmgren L, McGuigan FE, Berglundh S, Westman K, Christensson A, Åkesson K. Declining Estimated Glomerular Filtration Rate and Its Association with Mortality and Comorbidity Over 10 Years in Elderly Women. *Nephron*. 2015;130(4):245-255. doi:10.1159/000435790
7. Wang M, Sun X, Ni L, et al. Comparative Performance of Creatinine-Based GFR Estimation Equations in Exceptional Longevity: The Rugao Longevity and Ageing Study. *Clin Interv Aging*. 2020;Volume 15(101273480):733-742. doi:10.2147/CIA.S250535
8. Beridze G, Vetrano DL, Marengoni A, Dai L, Carrero JJ, Calderón-Larrañaga A. Concordance and Discrepancies Among 5 Creatinine-Based Equations for Assessing Estimated Glomerular Filtration Rate in Older Adults. *JAMA Netw Open*. 2023;6(3):e234211. doi:10.1001/jamanetworkopen.2023.4211
9. Tarantini L, McAlister FA, Barbati G, et al. Chronic kidney disease and prognosis in elderly patients with cardiovascular disease: Comparison between CKD-EPI and Berlin Initiative Study-1 formulas. *Eur J Prev Cardiol*. 2016;23(14):1504-1513. doi:10.1177/2047487316638454
10. Van Pottelbergh G, Vaes B, Adriaensen W, et al. The glomerular filtration rate estimated by new and old equations as a predictor of important outcomes in elderly patients. *BMC Med*. 2014;12(1):27. doi:10.1186/1741-7015-12-27
11. Kuhn A, van der Giet M, Kuhlmann MK, et al. Kidney Function as Risk Factor and Predictor of Cardiovascular Outcomes and Mortality Among Older Adults. *Am J Kidney Dis*. 2021;77(3):386-396.e1. doi:https://dx.doi.org/10.1053/j.ajkd.2020.09.015

12. Fu EL, Carrero JJ, Sang Y, et al. Association of Low Glomerular Filtration Rate With Adverse Outcomes at Older Age in a Large Population With Routinely Measured Cystatin C. *Ann Intern Med*. 2024;177(3):269-279. doi:10.7326/M23-1138
